# *Tetrahymena* meiosis: Simple yet ingenious

**DOI:** 10.1371/journal.pgen.1009627

**Published:** 2021-07-15

**Authors:** Josef Loidl

**Affiliations:** Department of Chromosome Biology, Max Perutz Labs, University of Vienna, Vienna, Austria; The University of North Carolina at Chapel Hill, UNITED STATES

## Abstract

The presence of meiosis, which is a conserved component of sexual reproduction, across organisms from all eukaryotic kingdoms, strongly argues that sex is a primordial feature of eukaryotes. However, extant meiotic structures and processes can vary considerably between organisms. The ciliated protist *Tetrahymena thermophila*, which diverged from animals, plants, and fungi early in evolution, provides one example of a rather unconventional meiosis. *Tetrahymena* has a simpler meiosis compared with most other organisms: It lacks both a synaptonemal complex (SC) and specialized meiotic machinery for chromosome cohesion and has a reduced capacity to regulate meiotic recombination. Despite this, it also features several unique mechanisms, including elongation of the nucleus to twice the cell length to promote homologous pairing and prevent recombination between sister chromatids. Comparison of the meiotic programs of *Tetrahymena* and higher multicellular organisms may reveal how extant meiosis evolved from proto-meiosis.

## Introduction

Meiosis is a special type of cell division through which eukaryotic germ progenitor cells halve the somatic diploid chromosome set to generate the gametic haploid set for sexual reproduction. During this process, parental genomes are shuffled within the gametes, which increases genetic diversity in the offspring. Hallmarks of meiosis are the formation of programmed DNA double-strand breaks (DSBs), searching and pairing of homologous parental chromosomes, exchange of sections between homologous chromosomes, and segregation of the resulting mosaic chromosomes to haploid daughter cells. DNA ends at DSB sites can interact with intact DNA molecules to probe homology, pair, and initiate homology-dependent repair, which ultimately leads to interhomolog crossovers (COs) (see [1]). Homologous pairing usually culminates in the formation of a protein structure, the synaptonemal complex (SC), which intimately connects the homologs and appears to regulate CO formation (see [2]).

Initial research on meiosis focused on organisms that were suitable for cytological and genetic analysis. Later, the era of molecular biology shifted the focus to budding yeast as the model organism, which revealed molecular details of the exchange of homologous DNA sequences and the cellular machinery of chromosome segregation. In hindsight, the choice of yeast was a good one because many features of yeast meiosis turn out to be typical of canonical meiosis, which prevails in vertebrates and plants, and whose understanding is critical for clinical human genetics and plant breeding. However, from studies in fission yeast, *Drosophila*, and *Caenorhabditis elegans*, it became clear that details of the meiotic process can vary. Therefore, meiosis research had to include nonstandard model organisms, preferably from distant evolutionary lineages, in order to capture the full range of meiotic diversity and commonalities and to understand the origin of meiosis and its impact on eukaryotic evolution.

This is where *Tetrahymena thermophila* entered the stage. *Tetrahymena* is a ciliated protist of the kingdom Chromalveolata, which branched off early in evolution from animals, plants, and fungi. This versatile model organism rose to prominence based on its contribution to unraveling programmed DNA elimination, identifying the first cytoskeletal motor, dynein, and elucidating the role of histone acetylation in transcription, and, particularly, in the Nobel Prize–winning discoveries of self-splicing introns and telomere organization (see [3]). Early studies revealed some structural peculiarities of *Tetrahymena* meiosis. Since then, closer investigation has made *Tetrahymena* meiosis the best-studied model outside the classical fungal, plant, and animal models and uncovered notable deviations from canonical meiosis (see [4]). Understanding this uncommon and, in some ways, minimalistic meiosis may help us to understand many mysterious aspects of the meiotic process and the evolutionary basis of functional adaptations to the meiotic program that exist in higher eukaryotes.

## *Tetrahymena* sexual reproduction

**Fig 1:**
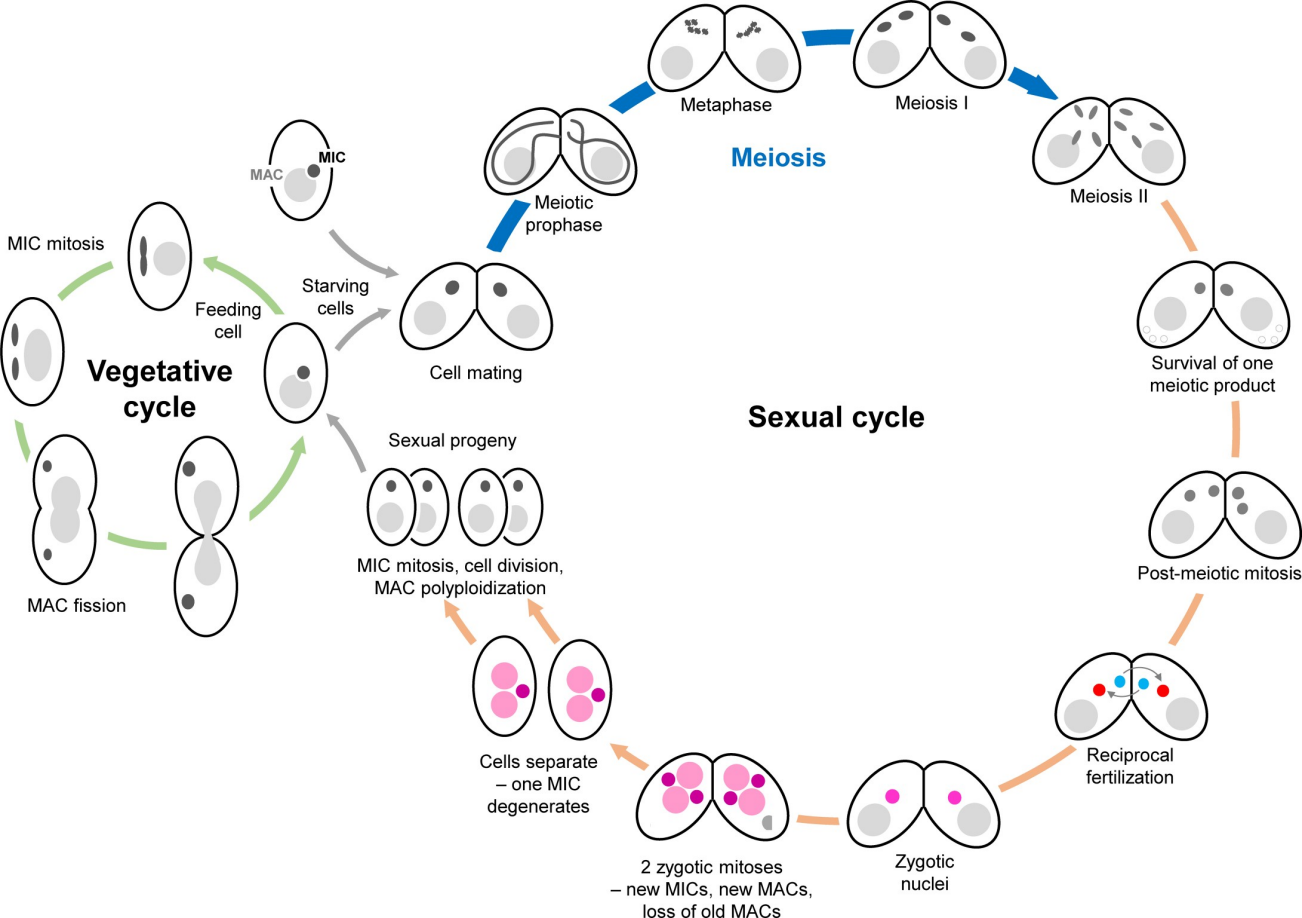
Vegetative and sexual cycles of *Tetrahymena thermophila*. During vegetative growth (left), the 2 nuclei divide asynchronously. First, the germline nucleus (the MIC) divides mitotically, then the somatic nucleus (MAC) stretches and splits amitotically, and, finally, the cleavage furrow closes to divide the cell. Meiosis is induced when starved cells of 2 different mating types meet (right). The cells mate (“conjugate”), and their MICs undergo synchronous meioses. Three of the 4 meiotic products are degraded, while the remainder is selected and recruited to the conjugation junction, where it undergoes a postmeiotic mitosis. This division produces 2 gametic nuclei, one migratory and the other stationary. Migratory nuclei are exchanged between the partner cells and fertilize their stationary nuclei. The resulting genetically identical zygote nuclei divide twice, and the daughter nuclei differentiate into the new MICs and MACs of 4 sexual progeny cells, while the old MAC is eliminated via autophagy. As soon as new MACs form, they start to polyploidize. The 5 MIC chromosomes are fragmented into about 200 MAC chromosomes, and about one-third of the MIC genome is eliminated by the removal of thousands of internal eliminated sequences (see [76]). MAC, macronucleus; MIC, micronucleus.

*T*. *thermophila* can reproduce both vegetatively and sexually (Fig 1). This makes it, like the yeasts, convenient for studying meiotic mutants because meiosis-incompetent lines can be maintained. *Tetrahymena* is a unicellular organism with the special feature of having 2 functionally distinct nuclei per cell. The macronucleus (MAC) represents the soma, where all gene transcription occurs. It is highly polyploid (approximately 50×), divides by amitotic splitting, and becomes degraded during sexual reproduction (see [5]). In contrast, the micronucleus (MIC), containing 2n = 10 roughly metacentric chromosomes, is genetically silent except for the transcription of noncoding RNA during sexual reproduction (see [6]). The MIC functions as the germline: During vegetative growth, it divides mitotically, and during sexual reproduction, it undergoes meiosis and produces gametic nuclei.

Notably, *Tetrahymena* has 7 sexes. During development of the somatic nucleus, sex is determined by the random cleavage and joining of DNA segments among gene pairs at the germline mating type locus [7]. Mating can occur between cells of any 2 different sexes, which increases the chance of bumping into a mating partner to 6 out of 7 compared with a mere 50% in species with 2 sexes. Sexual reproduction begins when starved cells of complementary sexes meet. They first enter the so-called costimulation stage, in which they sense the presence of cells of a different mating type (see [8]). These cells can mate (“conjugate”) and initiate meiosis within 2 hours and complete it within 5 hours [9]. The need for starvation to gain sexual competence is surprising. For yeast, this strategy is understandable because meiosis produces hardy spores that can outlast lean times. For *Tetrahymena*, whose meiotic products are not long lasting, we can only suppose that they adjourn meiosis until an otherwise unproductive period when vegetative reproduction is halted, anyway. Alternatively, cells might perceive nutritional stress as dwindling vigor owing to the accumulation of mutations and respond by genome rejuvenation via sex (see [10,11]).

**Fig 2:**
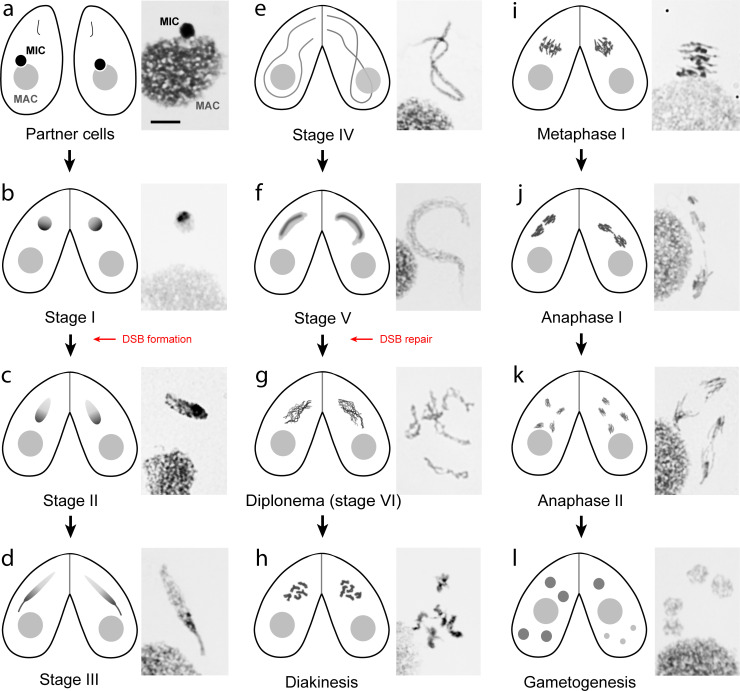
*Tetrahymena* meiotic stages. Schematic diagrams of mating cells and microscopy images of Giemsa-stained meiotic nuclei. Meiotic prophase is staged according to [79]. (**a)** In nonmeiotic cells, the MIC is located in a pocket on the MAC surface. (**b)** In stage I conjugating cells, the round MIC moves away from the MAC. The progress of meioses in conjugated cells is largely synchronous. (**c–e)** Once DSBs begin to form, the MICs start stretching and elongate to about twice the length of the cell. (**f)** MICs then shorten, and all DSBs are repaired by the end of stage V. (**g)** Progressive chromatin compaction reveals the formation of 5 bivalents. (**h)** Bivalents with protruding centromeres have reached maximal condensation. (**i)** Bivalents arranged in a metaphase plate. (**j, k)** Anaphase I and II follow the conventional scheme. (**l)** After telophase II (left cell), one of the 4 meiotic products is selected to divide into 2 gametic nuclei, whereas the other 3 products degenerate (right cell). Bar: 10 μm. DSB, double-strand break; MAC, macronucleus; MIC, micronucleus.

Meiotic progression can be easily followed and staged (Fig 2) [5]: During prophase, nuclei stretch into thin threads, and cytological and molecular markers of DSB formation and processing appear [12]. Chromosome pairing takes place without an SC [13,14]. At the end of prophase, nuclei shorten and at diplotene/diakinesis, condensed bivalents become discernible. This is followed by closed first and second meiotic divisions (Fig 2). The reciprocal fertilization of gametic pronuclei of 2 mating cells produces zygotic nuclei, which generate new germline and somatic nuclei (Fig 1). Meiotic genes show a characteristic temporal transcription pattern, with maximal expression in mating cells occurring from as early as 2 hours after mixing meiosis-competent cells [15]. Interestingly, some meiotic genes start being expressed at the costimulation stage, prior to pairing between mating partners (see NCBI dataset GSE132677; [8]). Although at this point cells may return to the vegetative cycle if the nutritional situation improves, such preparedness will speed up the mating process if starvation persists.

Its particular proficiency in harnessing ubiquitous proteins for meiotic functions allows *Tetrahymena* to employ comparatively few meiosis-specific genes. Studies of deletion mutants showed that of more than 100 genes with an expression peak at the early mating stage, only about one-quarter could be assigned a function in meiotic pairing and/or recombination (Table 1). Another quarter are involved in the maturation of sexual progeny, and about 10% are regulatory (encode transcription factors, cyclins or cyclin-dependent kinases, or control meiosis and other mating processes) [16–21]. Deletion of the remaining genes produced no apparent defect—either they are redundant or their loss causes only moderate meiotic nondisjunction. In fact, *Tetrahymena* meiosis is remarkably error tolerant: Even nullisomic gametic nuclei may be fertile because missing chromosomes can be supplied to the zygote by the mating partner, and selective replication can correct chromosomal imbalances in progeny somatic nuclei. However, germline nuclei will accumulate aneuploidies over multiple vegetative reproduction cycles, leading to decreasing fertility in subsequent generations.

**Table 1:** Genes with functions in *Tetrahymena* meiosis.

Process TGD ID	NCBI Gene ID, Protein ID	Meiosis specific	Function	Budding yeast homolog	Reference(s)
**Meiosis initiation and regulation**					
* TCDK3* (TTHERM_00483640)	Gene ID: 7840816, XP_001017477.2	Y	Meiotic cyclin–dependent kinase	*CDC28*	[17]
* CYC2* (TTHERM_00079530)	Gene ID: 7831246, XP_001015793.1	Y	Meiotic B-type cyclin	*CLB4*	[16]
* CYC17* (TTHERM_00693080)	Gene ID: 7827170, XP_001023573.2	Y	Meiotic B-type cyclin	*CLB2*	[20]
* CYC28* (TTHERM_00082190)	Gene ID: 7845854, XP_001012564.3	Y	Meiotic cyclin	*—*	[21]
* E2FL1* (TTHERM_00695710)	Gene ID: 7837814, XP_001025582.2	Y	Meiotic E2F family transcription factor	*—*	[18]
* DPL2* (TTHERM_00047010)	Gene ID: 7844987, XP_001014696.1	Y	Meiotic transcription factor	*—*	[19]
* APRO1* (TTHERM_00112830)	Gene ID: 7843544, XP_001010678.3	Y	Meiotic transcription factor	*—*	Unpublished
**DSB formation**					
* SPO11* (gene_000008668)^1^	Not listed	Y	Meiotic DSB formation	*SPO11*	[25]
* PARS11* (TTHERM_00133730)	Gene ID: 7829243, XP_001019652.3	Y	Meiotic DSB formation and control	(*REC114* ?)^3^	[41]
* PARS-L* (TTHERM_00530039)	Gene ID: 24442767, XP_001470656.1	Y	Meiotic DSB formation	*—*	Unpublished
**Homologous pairing**					
* CNA1* (TTHERM_00146340)	Gene ID: 7840155, XP_001011273.1	N	Centromeric histone, meiotic nuclear reorganization	*CSE4*	[27]
* MELG1* (TTHERM_00711850)	Gene ID: 7827803, XP_001025845.2	Y	Meiotic nuclear reorganization	*—*	[23]
* MELG2* (TTHERM_00289290)	Gene ID: 7846875, XP_001018646.3	Y	Meiotic nuclear reorganization	*—*	[23]
* MELG3* (gene_000025916)^1^	QPL17970.1	Y	Meiotic nuclear reorganization	*—*	[23]
**Crossover, chiasma formation**					
* ATR1* (TTHERM_01008650)	Gene ID: 7843404, XP_001026698.2	N	Meiotic DSB sensing	*MEC1*	[24]
* MRE11* (TTHERM_00721450)	Gene ID: 7841076, XP_001031877.2	N	DSB end processing	*MRE11*	[35,42]
* COM1* (TTHERM_00460480)	Gene ID: 7824714, XP_001018744.1	Y	DSB end processing	*COM1/SAE2*	[35]
* EXO1* (TTHERM_01179960)	Gene ID: 7846424, XP_001030028.2	(Y)^2^	DSB end processing	*EXO1*	[42]
* SGS1* (TTHERM_01030000)	Gene ID: 7824842, XP_001015163.2	N	Helicase, processing of recombination intermediates	*SGS1*	[48]
* MUS81* (TTHERM_00624870)	Gene ID: 7831680, XP_001022614.2	N	Double Holliday junction resolution	*MUS81*	[48]
* MMS4* (TTHERM_00194130)	Gene ID: 7844641, XP_001017166.1	N	Partner of Mus81, double Holliday junction resolution	*MMS4*	[48]
* DMC1* (TTHERM_00459230)	Gene ID: 7828368, XP_001024231.1	Y	Strand exchange with homologous chromosome	*DMC1*	[36]
* RAD51* (TTHERM_00142330)	Gene ID: 7827499, XP_001011071.1	N	Regulates Dmc1 nucleoprotein filament formation	*RAD51*	[36]
* HOP2* (TTHERM_00794620)	Gene ID: 7825538, XP_001020981.3	Y	Bivalent formation	*HOP2*	[25]
* MND1* (gene_000003690)^1^	Not listed	Y	Bivalent formation	*MND1*	[25]
* BIME2* (TTHERM_00530659)	Gene ID: 7827388, XP_001470665.1	Y	Promotes or stabilizes Dmc1 nucleoprotein filaments	(*RAD54* ?)^3^	[44]
* MSH4* (TTHERM_00857890)	Gene ID: 7832209, XP_001021931.2	Y	MutS-domain protein, stabilizes joint molecules	*MSH4*	[37]
* MSH5* (gene_000007168)^1^	Not listed	Y	MutS-domain protein, stabilizes joint molecules	*MSH5*	[37]
* ZHP3* (TTHERM_00049220)	Gene ID: 7830970, XP_001014817.4	Y	Stabilizes D-loop?	(*ZIP3* ?)^3^	[43]
* BIME1* (TTHERM_00460720)	Gene ID: 7825705, XP_001018768.3	Y	Bivalent formation	*—*	[43]
* MCMD1* (TTHERM_01207610)	Gene ID: 7836088, XP_001021907.2	Y	MCM domain protein, chiasma formation	*—*	[49]
* PAMD1* (TTHERM_001295283)	XM_012798783.1	Y	Partner of Mcmd1, chiasma formation		[49]
**Meiotic divisions**					
* REC8* (TTHERM_00245660)	Gene ID: 7841491, XP_001023795.2	N	Universal kleisin component of the cohesin complex	*SCC1*, *REC8*	[59]
* ESP1* (TTHERM_00297160)	Gene ID: 7834521, XP_001013249.2	N	Separase	*ESP1*	[59]

TTHERM_—identifier of annotated genes in the TGD (http://ciliate.org/).

^1^Incorrect annotation in TGD; the transcript identifier (gene_) from the TFGD (http://tfgd.ihb.ac.cn/) is shown instead.

^2^*EXO1* has a nonessential function in vegetative cells.

^3^Homology doubtful.

DSB, double-strand break; TFGD, *Tetrahymena* Functional Genomics Database; TGD, *Tetrahymena* Genome Database.

## Nuclear reorganization and chromosome pairing

The most visually striking stage in *Tetrahymena* meiosis is elongation of the prophase nucleus to about twice the cell length (Fig 2) [22,23]. This requires *SPO11*-dependent programmed DSBs and the DNA damage sensor kinase ATR [24]. Artificial DNA damage can restore meiotic nuclear elongation in *spo11* mutants [25], showing that elongation is triggered by a DNA damage signal. Nuclear elongation is driven by the polymerization of intranuclear microtubules [13,24,26], aided by elements of the cytoskeleton, membrane-associated proteins, and a set of microtubule-associated motor proteins. However, identifying the specific proteins involved in nuclear elongation is notoriously difficult because they are probably also used in other essential processes, so inhibiting them would impede cell growth and motility. However, it has been possible to identify a few meiosis-specific factors that repurpose the microtubule apparatus for meiotic nuclear reorganization [23].

**Fig 3:**
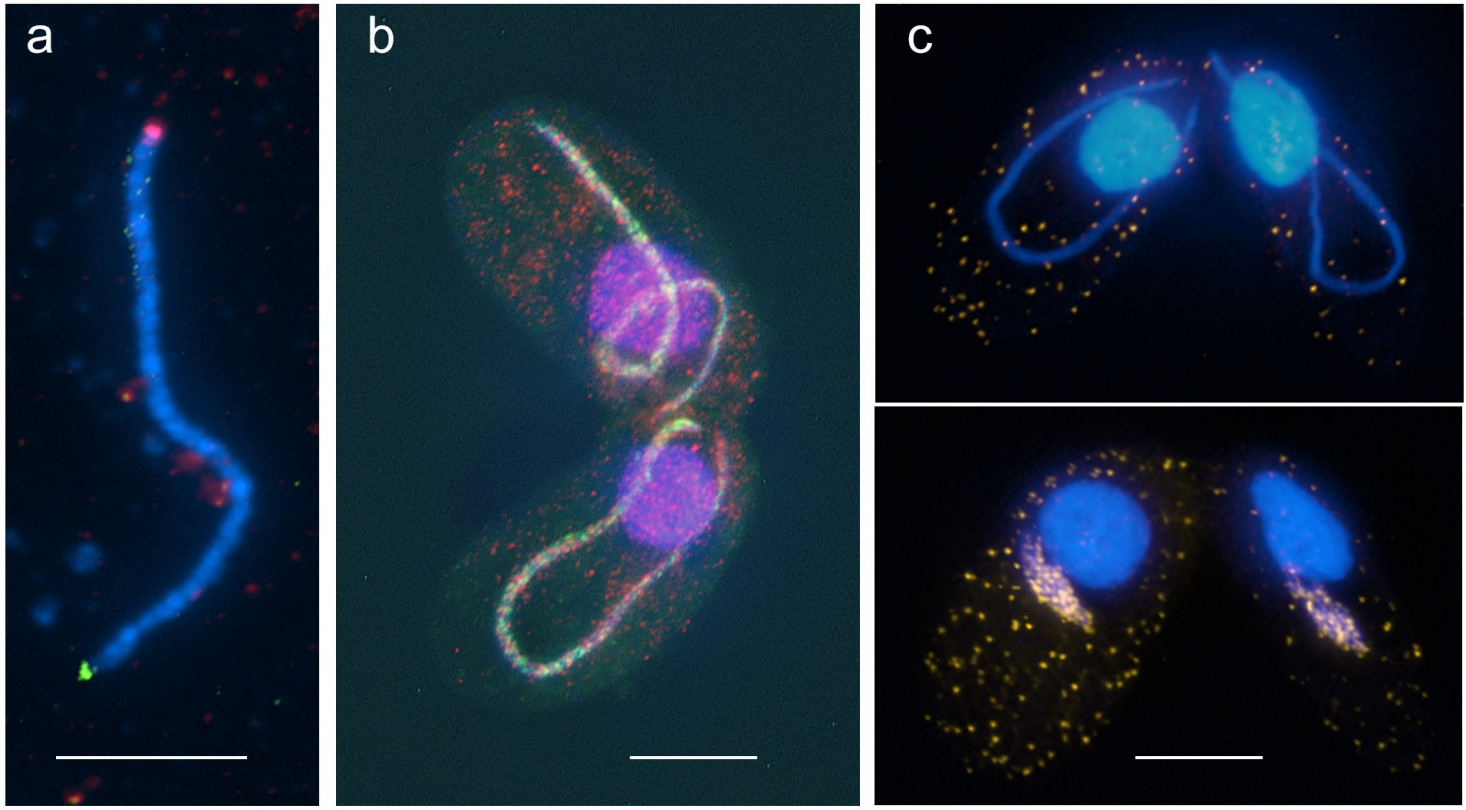
Examples of mating cells and meiotic nuclei. **(a)** An isolated meiotic nucleus showing the polarized arrangement of chromosome arms with centromeres (immunostained for centromere protein Cna1, red) and telomeres (fluorescence in situ hybridization with a telomere-repeat probe, green) attached to opposite ends. **(b)** Mating cells immunostained for the meiosis-specific recombination protein Dmc1 (green) and its ubiquitous paralog Rad51 (red). Elongated meiotic nuclei only show Dmc1, whereas the somatic nuclei only show Rad51. (**c)** Meiotic cells with fully elongated (top) and shortening (bottom) nuclei incubated with BrdU. BrdU incorporation (yellow) into shortening nuclei only indicates that DSB repair occurs at this stage. Bars: 10 μm. BrdU, bromodeoxyuridine; DSB, double-strand break.

Within the elongated nucleus (often referred to as the crescent), chromosome arms are oriented in parallel, with centromeres and telomeres attached to opposite ends (Fig 3) [27,28]. This arrangement is believed to promote the juxtapositioning of homologous regions, and, thereby, homologous pairing and CO [27,29]. If elongation is prevented by inhibiting microtubules, DSBs are repaired by recombinational repair via the sister chromatid [24]. Thus, the dual role of Spo11 in inducing DSBs and nuclear elongation ensures that programmed DSBs can be repaired via the homologous chromosome.

Unlike the horsetail movement of fission yeast, which disentangles chromosomes and sorts them by size (see [30]), live cell imaging showed that nuclear elongation is not accompanied by twisting or meandering movements [31]. Thus, the parallel orientation of chromosome arms appears sufficient to align homologous regions. While this arrangement resembles the meiotic chromosome bouquet found in most eukaryotes [32], homologs of Sun1, KASH-domain proteins, and other conserved bouquet-related proteins have not been identified in *Tetrahymena* [27]. Therefore, it is likely that the polarized prophase nucleus represents an analogous device rather than an extreme version of the classical bouquet [23]. Interestingly, successful chromosome pairing and crossing over depend more on centromere clustering than on telomere clustering [23]. Given the increasingly recognized importance of centromeres in meiotic pairing in a variety of species (see [23,27]), it is conceivable that centromere clustering (which is a consequence of the anaphase orientation of chromatids in nuclear division) may have facilitated homologous chromosome alignment in proto-meiosis prior to the evolution of telomere-driven mechanisms for alignment, such as the bouquet.

Nuclear elongation is also found in some other ciliates [4,33]. Yet others use a so-called parachute structure, in which chromatin is unevenly distributed in a drop-shaped nucleus; this structure is more reminiscent of the classic bouquet (see [33,34]). Some ciliates appear to lack an SC, whereas others possess axial element-like structures or even stretches of SC-like material or fragmentary SCs, and normal SCs are present in other chromalveolates [4,14]. Thus, there seems to be a tendency within the ciliate clade toward loss of the SC, which appears to correlate with the presence of elongated meiotic nuclei. This inverse association supports the notion that the spatial confinement of chromosomes within a tubular nucleus substitutes for physical linkage of the homologs by an SC [27].

## Meiotic recombination

The reciprocal exchange of broken DNA strands enables interhomologous COs to form. In this process, numerous DSBs are generated by Spo11 to ensure proper homology searching and homologous pairing (see [1]). In *Tetrahymena*, DSB formation and repair have been detected by the transient appearance of chromosome fragments in pulsed field electrophoresis gels [35], by localization of the recombination protein Dmc1 [36], and by bromodeoxyuridine (BrdU) incorporation during the recombinational repair of DSBs (Fig 3) [27]. Approximately 200 programmed DSBs are formed during *Tetrahymena* meiosis [37]. Early steps in meiotic recombination follow the canonical pathway, with Spo11 inducing DSBs. However, a homolog of TOPOVIBL, which is an essential complex partner of Spo11 in other species [38], seems to be missing or has diverged beyond recognition in *Tetrahymena*. As in yeast, animals, and plants, poorly conserved cofactors (see [39,40]) are also needed. These include Pars11, which functions in DSB formation and control (analogous to yeast Rec114; [41]) and an as yet uncharacterized orphan protein encoded by gene TTHERM_00530039 (Table 1).

A DSB can be repaired in various ways. There is a choice between nontemplated repair, such as nonhomologous end-joining (NHEJ), and repair using a homologous DNA molecule from the sister chromatid or homologous chromosome as the template. NHEJ is unlikely to play even a backup role in *Tetrahymena* meiotic DSB repair [12]. Therefore, the vast majority, if not all, of meiotic DSBs are repaired via homologous recombination. Once DSBs have formed, flanking DNA tracts are resected to expose 3′ single-stranded tails capable of invading double-stranded DNA. The usual suspects are involved in dealing with *Tetrahymena* DSBs: the endo/exonuclease Mre11, the exonuclease Exo1, and Sae2 (Com1) (Table 1). However, an as yet unknown nuclease that initiates DSB processing must also exist because single-stranded tracts can be generated in the absence of Mre11 [42]. Like most model organisms, *Tetrahymena* requires for strand exchange the ubiquitous Rad51 recombinase and its meiosis-specific paralog Dmc1, which associate as multimeric nucleofilaments with single-stranded tracts to enable their interaction with double-stranded DNA. A unique aspect of this stage in *Tetrahymena* is that Dmc1 forms numerous foci in meiotic prophase nuclei, whereas Rad51 is not detectable, despite its abundance at vegetative DNA damage repair sites. This lack is interpreted by Rad51 playing only a regulatory role in interhomolog recombination and only short Rad51-laden tracts being involved in intersister exchange [35,36]. A few other factors assist in converting DSBs into COs by stabilizing the DNA nucleofilament or promoting homologous strand invasion; some are clear orthologs of yeast proteins, such as Hop2 and Mnd1 [25], whereas others (Zhp3, Bime1, and Bime2) have no clear assignment [43,44] (Table 1).

For homologous recombination and segregation, DSB repair must be redirected from the sister chromatid, which is the spatially preferred template, to the homolog [45]. In budding yeast and other model organisms, activities of the Red1 and Hop1 chromosome axis proteins and the Mek1 kinase switch recombination from a mitosis-like intersister mode to a meiotic interhomolog mode (see [46]). This transition is gradual, as early DSBs are primarily repaired by the sister, whereas later in prophase, when DSBs become more abundant, the barrier to intersister exchange is enforced and interhomolog repair prevails [47]. In *Tetrahymena*, the situation is different, since DSBs are simultaneously repaired late in prophase [48]. Therefore, early intersister D loops are believed to be destabilized by the Sgs1 helicase in order to recycle DSBs for use in homologous recombination once homologs have become aligned [48]. In addition, Mcmd1 (a meiosis-specific minichromosome maintenance-like protein) and its partner Pamd1 help delay DSB repair to prevent precocious intersister recombination [49]. Nevertheless, the meiotic interhomolog bias may be weaker than in other organisms: Perfect repair (instead of meiosis failure) occurs in the absence of Dmc1 or Hop2, which are generally believed to promote interhomolog recombination [25,36], suggesting a strong potential for intersister repair. In addition, an unknown percentage of DSBs is likely channeled into nonreciprocal interhomolog recombination (conversion) events, which explains the huge discrepancy between the estimated numbers of DSBs (approximately 200) and putative chiasmata (approximately 20 to 40) [23,43].

In most eukaryotes, the SC is a prominent structure of meiotic prophase. It promotes the maturation of recombination intermediates into COs, and the SC or its lateral elements are believed to be involved in CO interference (the mutual suppression of neighboring COs) (see [50,51]) and in restricting the number of DSBs [52]. Although *Tetrahymena* does not possess a canonical SC, a nonconserved recombination protein, Bime1 (Sa15), forms thread-like structures on chromatin. This suggests that Bime1 may form part of or be arranged along an axial structure [43] that resembles SC lateral elements or the linear elements of fission yeast. Confirmation of such a meiotic chromosome scaffold would support the generality of the loop–axis model. According to this model, DSBs may be formed in chromatin loops, but repair requires the context of chromosome axes to which recombination proteins are attached [53,54].

Most eukaryotes use 2 major pathways to form COs: One is the class I pathway, which involves SC formation and ZMM proteins and generates COs that are subject to interference. The other is the class II pathway, which is largely ZMM independent and produces noninterfering COs [55]. In contrast, *Tetrahymena* uses a single mixed pathway that involves some ZMM proteins and the class II Holliday junction resolvase Mus81 [37,43]. Such limitation to a single CO pathway is another hallmark of *Tetrahymena*’s pared-down meiosis. Since *Tetrahymena* shares with fission yeast the absence of both an SC and the class I CO pathway, and since CO interference is weak in this organism [56,57], the question arises of whether *Tetrahymena*’s COs are interfering. However, without clear data on CO numbers and distribution or genetic map size (see [58]), neither this question nor the related question of whether the SC has a role in CO interference can be answered at present.

## Meiotic divisions and segregation

*Tetrahymena* undergoes closed meiotic divisions, i.e., the spindle apparatus is formed within the intact nuclear membrane [5]. The first division begins when nuclei have shortened and bivalents are arranged in a metaphase I plate at the nuclear equator. This requires reorganization of the microtubule apparatus from a unipolar arrangement to drive nuclear elongation at prophase to a bipolar arrangement to form the division spindle. Microtubule reorganization is coordinated by the meiosis-specific cyclin, Cyc28. In a *cyc28* deletion mutant, the monopolar microtubules prematurely undergo reorganization into a bipolar spindle, and chromosomes start to separate before homologs have paired. Consequently, chromosome segregation is irregular [21].

In *Tetrahymena*, the first and second meiotic divisions follow the canonical scheme with the segregation of homologs and sister chromatids, respectively. However, here, we encounter another unconventional aspect of *Tetrahymena* meiosis, namely the lack of a specialized meiotic cohesin [59]. Most eukaryotes possess a somatic and a meiotic version of the cohesin complex. This has been explained by meiotic cohesin having a specific function in the stepwise release of cohesion from chromosome arms and centromeres to allow homologs and sisters to separate in meiosis I and II, respectively [60]. In general, meiotic cohesin complexes are loaded during premeiotic S-phase. However, *Tetrahymena* has no chance to load a meiosis-specific cohensin, since it does not undergo dedicated premeiotic DNA synthesis [59,61,62]: Most starving cells are in G_2_ and enter meiosis or resume mitosis depending on whether they encounter a mating partner or are provided food. Notably, cohesin is absent in the somatic nucleus, where all gene transcription occurs. Thus, whereas in most eukaryotes, cohesin may be involved in the formation of topologically associated domains (TADs), which have been implicated in transcriptional regulation [63,64], this is not the case in *Tetrahymena*. In fact, Hi-C experiments have confirmed that somatic chromatin lacks TADs [28]. A possible explanation for the absence of this gene regulatory function in *Tetrahymena* is that the small size of its somatic chromosomes, with each containing only a few genes, may make elaborate regulatory mechanisms dispensable. Return-to-growth experiments and knockout studies have shown that, in principle, a single (meiotic) cohesin version is also sufficient for vegetative and meiotic divisions in budding yeast, (see [59,65]). This suggests that the vegetative version of cohesin found in most organisms is likely to be an optimized solution for gene regulation, which *Tetrahymena* does not need.

## The end game

Once 4 haploid meiotic products have formed in each of the 2 conjugating partners, they undergo a replication step whereupon 3 nuclei degenerate. This seems wasteful but may possibly allow for a quality control mechanism to select a healthy nucleus for survival. The selected nucleus undergoes a postmeiotic (PM) mitosis to produce 2 gametic pronuclei.

At this stage, another round of programmed DSBs is believed to take place [66]. These PM DSBs are induced by a topoisomerase II paralog with exclusive pronuclear expression, possibly assisted by Spo11. PM DSBs are proposed to be responsible for modified histone replacement during the reprogramming of generative to undifferentiated progeny nuclei since their repair is concomitant with the incorporation of newly synthesized histone H3 into pronuclei. Given the danger of DSBs to genome integrity, their usage in chromatin remodeling in *Tetrahymena* is remarkable but may parallel the functions of self-inflicted DSBs in transcriptional regulation and chromatin decondensation in other organisms [67–70].

The 2 identical gamete pronuclei of each cell then cross-fertilize with their counterparts in the partner cell. In this way, a pair of conjugating cells produces 4 progeny cells with identical germline genomes (Fig 1). This is another curious feature of *Tetrahymena* sexual reproduction, which at first sight seems to violate the postulate that meiosis should produce genetically, and, hence, phenotypically diverse siblings to avoid wasteful sibling competition [71,72]. However, siblings differentiate phenotypically due to random genotypic assortment of their polyploid somatic genomes. This means that over time, progeny cells that started with a 50:50 allele ratio for a somatic gene will acquire different allele ratios and may eventually become homozygous for either allele. In addition, imprecise somatic DNA elimination can create diversity in the genetic makeup of siblings (see below and [6]). Notably, inbreeding of siblings is largely prevented by the sexual immaturity of young sexual progeny. Only after approximately 85 to 90 vegetative fissions can cells mate with cells of a similar clonal age (see [73]). By this time, siblings have dispersed and are unlikely to encounter each other to mate [74].

## Mendelian and RNA-guided transgenerational epigenetic inheritance take place concomitantly in the germline

*Tetrahymena* and other ciliates have evolved the ultimate way of combating transposable elements (TEs) and other undesired DNA sequences, namely their elimination [75]. In *Tetrahymena*, the approximately 12,000 internal eliminated sequences (IESs; corresponding to about one-third of the genome) present in the germline nucleus are removed from the somatic nucleus, and somatic chromosomes are fragmented into approximately 225 minichromosomes [76]. The vast majority of IESs (approximately 18 MB) are TE-related sequences; the remainder are centromeres and perhaps other structural or regulatory elements that are dispensable for the function of the somatic nucleus [75–77]. Elimination of most germline-limited DNA sequences is guided by noncoding RNA, which is transcribed in the early meiotic nucleus [78]. This is the only detectable transcriptional activity of the germline nucleus, and it occurs concomitantly with meiotic recombination [79]. The transcription of small RNA precursors from both DNA strands produces double-stranded RNA molecules (see [6]), which are cleaved into small (28 to 30 nucleotides) RNAs (also called scan RNAs (scnRNAs)) [80]. scnRNAs are transferred from the germline nucleus to the old somatic nucleus, where they undergo a selection process: Those that match sequences in the somatic nucleus are degraded, and the remaining scnRNAs (corresponding to germline-limited sequences) enter the new somatic nucleus, where they instruct heterochromatinization and IES elimination [6]. Thus, 2 layers of sexual inheritance exist in *Tetrahymena*: One is mendelian inheritance via meiosis and fertilization of the germline nucleus, and the other is the transfer to sexual progeny of RNA-encoded information on the genetic makeup of the parental somatic nucleus. Notably, this information flow is not flawless as DNA elimination is sometimes imprecise, and some sequences are erroneously retained or eliminated. Such sequence variants may become homozygous in the polyploid somatic nucleus due to random assortment during amitotic segregation, allowing cells to adapt to environmental changes. Once fixed in the somatic nucleus, advantageous variants could be inherited by sexual progeny via selected scnRNA [6,75].

Compared with its modest genetic commitment to meiosis, *Tetrahymena* expends considerable effort in eliminating TEs from the soma: Of all genes that are exclusively or preferentially expressed within the first 2 hours after the initiation of mating, about one-quarter are involved in IES elimination. But why doesn’t *Tetrahymena* instead remove these sequences from its germline to prevent their transmission to progeny? One answer may be a benefit of TE activity in rearranging the germline genome or creating novel genes, thereby providing opportunities for evolutionary innovation (see [81] and lit. cit. therein).

The massive burst of genome-wide transcriptional activity concomitant with early meiosis raises the question of whether these processes influence each other. Efforts are currently underway to map DSBs and determine whether scnRNA transcription and meiotic DSB formation avoid spatial overlap or, in contrast, share open chromatin tracts such as nucleosome-depleted regions at transcription start sites (see [40]). In this regard, *Tetrahymena* is an ideal model to study functional relationships between recombination and transcription. Owing to its nuclear dualism, all transcription in the meiotic nucleus is nonessential and can be tweaked or even shut down completely without serious consequences for the progression of meiosis.

## Conclusions

*Tetrahymena* sexual reproduction has some unusual features. It has 7 sexes, and pairs of mating cells produce genetically identical progeny. In addition to transmission of genetic information via meiosis and fertilization, there is RNA-encoded epigenetic transgenerational inheritance of the genetic constitution of the soma. Most notably, the meiotic step in the reproduction cycle has some unique features. For example, it lacks the elaborate meiosis-specific SC and, instead, ensures homologous pairing via nuclear elongation and largely utilizes genes that have a dual function in somatic and meiotic chromosome dynamics. Combined with the almost exclusive use of somatic repair genes for its streamlined CO pathway and a single ubiquitous cohesin complex, this allows the organism to capitalize on a small number of meiosis-specific genes. Unlike most eukaryotes, which have evolved increasingly diverse and sophisticated meioses, ciliated protists have simplified the process, with *Tetrahymena* probably being the most pared-down example. Therefore, although not primordial, *Tetrahymena*’s meiosis, stripped down to the essentials of the process, may mirror the minimal features of a hypothetical proto-meiosis.

## References

[1] Keeney S. Spo11 and the formation of DNA double-strand breaks in meiosis. In: Lankenau D-H, Egel R, editors. Recombination and Meiosis. Berlin, Heidelberg: Springer-Verlag; 2008. p. 81–123. 10.1007/7050_2007_026 PMC3172816 21927624

[2] Zickler D. From early homologue recognition to synaptonemal complex formation. Chromosoma. 2006;115:158–74. doi: 10.1007/s00412-006-0048-6 16570189

[3] Ruehle MD, Orias E, Pearson CG. *Tetrahymena* as a unicellular model eukaryote: genetic and genomic tools. Genetics. 2016;203:649–65. doi: 10.1534/genetics.114.169748 27270699 PMC4896184

[4] Loidl J. Conservation and variability of meiosis across the eukaryotes. Annu Rev Genet. 2016;50:293–316. doi: 10.1146/annurev-genet-120215-035100 27686280

[5] Cole E, Sugai T. Developmental progression of *Tetrahymena* through the cell cycle and conjugation. In: Collins K, editors. Tetrahymena thermophila. San Diego:Academic Press; 2012. p. 177–236. 10.1016/B978-0-12-385967-9.00007-4 22444146

[6] Noto T, Mochizuki K. Whats, hows and whys of programmed DNA elimination in *Tetrahymena*. Open Biol. 2017;7:rsob.170172. doi: 10.1098/rsob.170172 29021213 PMC5666084

[7] Cervantes MD, Hamilton EP, Xiong J, Lawson MJ, Yuan D, Hadjithomas M, et al. Selecting one of several mating types through gene segment joining and deletion in *Tetrahymena thermophila*. PLoS Biol. 2013;11:e1001518. doi: 10.1371/journal.pbio.1001518 23555191 PMC3608545

[8] Ma Y, Yan G, Han X, Zhang J, Xiong J, Miao W. The sexual cell cycle initiation is regulated by CDK19/CYC9 in *Tetrahymena thermophila*. J Cell Sci. 2020;133:jcs235721. doi: 10.1242/jcs.235721 32041901

[9] Martindale DW, Allis CD, Bruns PJ. Conjugation in *Tetrahymena thermophila*. A temporal analysis of cytological stages. Exp Cell Res. 1982;140:227–36. doi: 10.1016/0014-4827(82)90172-0 7106201

[10] Bernstein C. Why are babies young? Meiosis may prevent aging of the germ line. Perspect Biol Med. 1979;22:539–44. doi: 10.1353/pbm.1979.0041 573881

[11] MacPherson B, Scott R, Gras R. Sex and recombination purge the genome of deleterious alleles: an individual based modeling approach. Ecol Complex. 2021;45:100910.

[12] Loidl J, Lorenz A. DNA double-strand break formation and repair in *Tetrahymena* meiosis. Semin Cell Dev Biol. 2016;54:126–34. doi: 10.1016/j.semcdb.2016.02.021 26899715

[13] Wolfe J, Hunter B, Adair WS. A cytological study of micronuclear elongation during conjugation in *Tetrahymena*. Chromosoma. 1976;55:289–308. doi: 10.1007/BF00292827 824106

[14] Chi J, Mahé F, Loidl J, Logsdon J, Dunthorn M. Meiosis gene inventory of four ciliates reveals the prevalence of a synaptonemal complex-independent crossover pathway. Mol Biol Evol. 2014;31:660–72. doi: 10.1093/molbev/mst258 24336924

[15] Xiong J, Lu X, Zhou Z, Chang Y, Yuan D, Tian M, et al. Transcriptome analysis of the model protozoan, *Tetrahymena thermophila*, using deep RNA sequencing. PLoS ONE. 2012;7:e30630. doi: 10.1371/journal.pone.0030630 22347391 PMC3274533

[16] Xu Q, Wang R, Ghanam AR, Yan G, Miao W, Song X. The key role of *CYC2* during meiosis in *Tetrahymena thermophila*. Protein Cell. 2016;7:236–49. doi: 10.1007/s13238-016-0254-9 27008457 PMC4818844

[17] Yan G, Zhang J, Shodhan A, Tian M, Miao W. Cdk3, a conjugation-specific cyclin-dependent kinase, is essential for the initiation of meiosis in *Tetrahymena thermophila*. Cell Cycle. 2016;15:2506–14. doi: 10.1080/15384101.2016.1207838 27420775 PMC5026800

[18] Zhang J, Tian M, Yan G, Shodhan A, Miao W. E2fl1 is a meiosis-specific transcription factor in the protist *Tetrahymena thermophila*. Cell Cycle. 2017;16:123–35. doi: 10.1080/15384101.2016.1259779 27892792 PMC5270522

[19] Zhang J, Yan GX, Tian M, Ma Y, Xiong J, Miao W. DP-like transcription factor protein interacts with E2fl1 to regulate meiosis in *Tetrahymena thermophila*. Cell Cycle. 2018;17:634–42. doi: 10.1080/15384101.2018.1431595 29417875 PMC5969552

[20] Yan G, Dang H, Tian M, Zhang J, Shodhan A, Ning Y, et al. Cyc17, a meiosis-specific cyclin, is essential for anaphase initiation and chromosome segregation in *Tetrahymena thermophila*. Cell Cycle. 2016;15:1855–64. doi: 10.1080/15384101.2016.1188238 27192402 PMC4968904

[21] Loidl J. Cyclin Cyc28 is required for the coordinated progression of meiosis in *Tetrahymena thermophila*. 2020. Available from: http://wwwsuprdborg/index php/experiment/details/SUPR000497.

[22] Ray C Jr. Meiosis and nuclear behaviour in *Tetrahymena pyriformis*. J Protozool. 1956;3:88–96.

[23] Tian M, Agreiter C, Loidl J. Spatial constraints on chromosomes are instrumental to meiotic pairing. J Cell Sci. 2020;133:jcs253724. doi: 10.1242/jcs.253724 33172984 PMC7725606

[24] Loidl J, Mochizuki K. *Tetrahymena* meiotic nuclear reorganization is induced by a checkpoint kinase-dependent response to DNA damage. Mol Biol Cell. 2009;20:2428–37. doi: 10.1091/mbc.e08-10-1058 19297526 PMC2675622

[25] Mochizuki K, Novatchkova M. Loidl J. DNA double-strand breaks, but not crossovers, are required for the reorganization of meiotic nuclei in *Tetrahymena*. J Cell Sci. 2008;121:2148–58. doi: 10.1242/jcs.031799 18522989 PMC3184542

[26] Kaczanowski A, Gaertig J, Kubiak J. Effect of the antitubulin drug nocodazole on meiosis and postmeiotic development in *Tetrahymena thermophila*. Induction of achiasmatic meiosis. Exp Cell Res. 1985;158:244–56. doi: 10.1016/0014-4827(85)90447-1 3996478

[27] Loidl J, Lukaszewicz A, Howard-Till RA, Koestler T. The *Tetrahymena* meiotic chromosome bouquet is organized by centromeres and promotes interhomolog recombination. J Cell Sci. 2012;125:5873–80. doi: 10.1242/jcs.112664 22976299

[28] Luo ZY, Hu TF, Jiang H, Wang RY, Xu QL, Zhang SM, et al. Rearrangement of macronucleus chromosomes correspond to TAD-like structures of micronucleus chromosomes in *Tetrahymena thermophila*. Genome Res. 2020;30:406–14. doi: 10.1101/gr.241687.118 32165395 PMC7111529

[29] Loidl J, Scherthan H. Organization and pairing of meiotic chromosomes in the ciliate *Tetrahymena thermophila*. J Cell Sci. 2004;117:5791–801. doi: 10.1242/jcs.01504 15522890

[30] Hiraoka Y, Dernburg AF. The SUN rises on meiotic chromosome dynamics. Dev Cell. 2009;17:598–605. doi: 10.1016/j.devcel.2009.10.014 19922865

[31] Howard-Till RA, Loidl J. Condensins promote chromosome individualization and segregation during mitosis, meiosis, and amitosis in *Tetrahymena thermophila*. Mol Biol Cell. 2018;29:466–78. doi: 10.1091/mbc.E17-07-0451 29237819 PMC6014175

[32] Scherthan H. A bouquet makes ends meet. Nat Rev Mol Cell Biol. 2001;2:621–7. doi: 10.1038/35085086 11483995

[33] Raikov IB. The protozoan nucleus. Morphology and evolution. Wien and New York: Springer-Verlag; 1982. p. 474.

[34] Devidé Z, Geitler L. Die Chromosomen der Ciliaten. Chromosoma. 1950;1947(3):110–36.

[35] Lukaszewicz A, Howard-Till RA, Novatchkova M, Mochizuki K, Loidl J. *MRE11* and *COM1*/*SAE2* are required for double-strand break repair and efficient chromosome pairing during meiosis of the protist *Tetrahymena*. Chromosoma. 2010;119:505–18. doi: 10.1007/s00412-010-0274-9 20422424

[36] Howard-Till RA, Lukaszewicz A, Loidl J. The recombinases Rad51 and Dmc1 play distinct roles in DNA break repair and recombination partner choice in the meiosis of *Tetrahymena*. PLoS Genet. 2011;7:e1001359. doi: 10.1371/journal.pgen.1001359 21483758 PMC3069121

[37] Shodhan A, Lukaszewicz A, Novatchkova M, Loidl J. Msh4 and Msh5 function in SC-independent chiasma formation during the streamlined meiosis of *Tetrahymena*. Genetics. 2014;198:983–93. doi: 10.1534/genetics.114.169698 25217051 PMC4224184

[38] Robert T, Nore A, Brun C, Maffre C, Crimi B, Bourbon HM, et al. The TopoVIB-Like protein family is required for meiotic DNA double-strand break formation. Science. 2016;351:943–9. doi: 10.1126/science.aad5309 26917764

[39] Cole F, Keeney S, Jasin M. Evolutionary conservation of meiotic DSB proteins: more than just Spo11. Genes Dev. 2010;24:1201–7. doi: 10.1101/gad.1944710 20551169 PMC2885656

[40] de Massy B. Initiation of meiotic recombination: how and where? Conservation and specificities among eukaryotes. Annu Rev Genet. 2013;47:563–99. doi: 10.1146/annurev-genet-110711-155423 24050176

[41] Tian M, Loidl J. A chromatin-associated protein required for inducing and limiting meiotic DNA double-strand break formation. Nucleic Acids Res. 2018;46:11822–34. doi: 10.1093/nar/gky968 30357385 PMC6294514

[42] Lukaszewicz A, Shodhan A, Loidl J. Exo1 and Mre11 execute meiotic DSB end resection in the protist *Tetrahymena*. DNA Repair (Amst). 2015;35:137–43. doi: 10.1016/j.dnarep.2015.08.005 26519827

[43] Shodhan A, Kataoka K, Mochizuki K, Novatchkova M, Loidl J. A Zip3-like protein plays a role in crossover formation in the SC-less meiosis of the protist *Tetrahymena*. Mol Biol Cell. 2017;28:825–33. doi: 10.1091/mbc.E16-09-0678 28100637 PMC5349789

[44] Shodhan A, Novatchkova M, Loidl J. *BIME2*, a novel gene required for interhomolog meiotic recombination in the protist model organism *Tetrahymena*. Chromosome Res. 2017;25:291–8. doi: 10.1007/s10577-017-9563-y 28803330 PMC5662671

[45] Borde V, de Massy B. Early DNA double-strand breaks pave the way for inter-homolog repair. Dev Cell. 2015;32:663–664. doi: 10.1016/j.devcel.2015.03.011 25805132

[46] Kim KP, Mirkin EV. So similar yet so different: the two ends of a double strand break. Mutat Res. 2018;809:70–80. doi: 10.1016/j.mrfmmm.2017.06.007 28693746

[47] Joshi N, Brown MS, Bishop DK, Börner GV. Gradual implementation of the meiotic recombination program via checkpoint pathways controlled by global DSB levels. Mol Cell. 2015;57:1–15. doi: 10.1016/j.molcel.2014.12.022 25661491 PMC4392720

[48] Lukaszewicz A, Howard-Till RA, Loidl J. Mus81 nuclease and Sgs1 helicase are essential for meiotic recombination in a protist lacking a synaptonemal complex. Nucleic Acids Res. 2013;41:9296–309. doi: 10.1093/nar/gkt703 23935123 PMC3814389

[49] Tian M, Loidl J. An MCM family protein promotes interhomolog recombination by preventing precocious intersister repair of meiotic DSBs. PLoS Genet. 2019;15:e1008514. doi: 10.1371/journal.pgen.1008514 31815942 PMC6922451

[50] Rog O, Köhler S, Dernburg AF. The synaptonemal complex has liquid crystalline properties and spatially regulates meiotic recombination factors. Elife. 2017;6:e21455. doi: 10.7554/eLife.21455 28045371 PMC5268736

[51] Lambing C, Kuo PC, Tock AJ, Topp SD, Henderson IR. ASY1 acts as a dosage-dependent antagonist of telomere-led recombination and mediates crossover interference in *Arabidopsis*. Proc Natl Acad Sci U S A. 2020;117:13647–58. doi: 10.1073/pnas.1921055117 32499315 PMC7306779

[52] Mu XJ, Murakami H, Mohibullah N, Keeney S. Chromosome-autonomous feedback down-regulates meiotic DNA break competence upon synaptonemal complex formation. Genes Dev. 2020;34:1605–18. doi: 10.1101/gad.342873.120 33184224 PMC7706706

[53] Panizza S, Mendoza MA, Berlinger M, Huang L, Nicolas A, Shirahige K, et al. Spo11-accessory proteins link double-strand break sites to the chromosome axis in early meiotic recombination. Cell. 2011;146:372–83. doi: 10.1016/j.cell.2011.07.003 21816273

[54] Lam I, Keeney S. Mechanism and regulation of meiotic recombination initiation. Cold Spring Harb Perspect Biol. 2014;7:a016634. doi: 10.1101/cshperspect.a016634 25324213 PMC4292169

[55] de los Santos T, Hunter N, Lee C, Larkin B, Loidl J, Hollingsworth NM. The Mus81/Mms4 endonuclease acts independently of double-Holliday junction resolution to promote a distinct subset of crossovers during meiosis in budding yeast. Genetics. 2003;164:81–94. 12750322 10.1093/genetics/164.1.81 PMC1462551

[56] Kohli J, Bähler J. Homologous recombination in fission yeast—absence of crossover interference and synaptonemal complex. Experientia. 1994;50:295–306. doi: 10.1007/BF01924013 8143803

[57] Smith GR, Nambiar M. New solutions to old problems: molecular mechanisms of meiotic crossover control. Trends Genet. 2020;36:337–46. doi: 10.1016/j.tig.2020.02.002 32294414 PMC7162993

[58] Lynch TJ, Brickner J, Nakano KJ, Orias E. Genetic map of randomly amplified DNA polymorphisms closely linked to the mating type locus of *Tetrahymena thermophila*. Genetics. 1995;141:1315–25. 8601476 10.1093/genetics/141.4.1315 PMC1206869

[59] Howard-Till RA, Lukaszewicz A, Novatchkova M, Loidl J. A single cohesin complex performs mitotic and meiotic functions in the protist *Tetrahymena*. PLoS Genet. 2013;9:e1003418. doi: 10.1371/journal.pgen.1003418 23555314 PMC3610610

[60] Petronczki M, Siomos MF, Nasmyth K. Un ménage à quatre: the molecular biology of chromosome segregation in meiosis. Cell. 2003;112:423–40. doi: 10.1016/s0092-8674(03)00083-7 12600308

[61] McDonald BB. The exchange of RNA and protein during conjugation in *Tetrahymena*. J Protozool. 1966;13:277–85. doi: 10.1111/j.1550-7408.1966.tb01908.x 5953847

[62] Doerder FP, Debault LE. Cytofluorometric analysis of nuclear DNA during meiosis, fertilization and macronuclear development in the ciliate *Tetrahymena pyriformis*, syngen 1. J Cell Sci. 1975;17:471–93. 806605 10.1242/jcs.17.3.471

[63] El Khattabi L, Zhao H, Kalchschmidt J, Young N, Jung S, Van Blerkom P, et al. A pliable Mediator acts as a functional rather than an architectural bridge between promoters and enhancers. Cell. 2019;178:1145–58. doi: 10.1016/j.cell.2019.07.011 31402173 PMC7533040

[64] Robson MI, Ringel AR, Mundlos S. Regulatory landscaping: how enhancer-promoter communication is sculpted in 3D. Mol Cell. 2019;74:1110–22. doi: 10.1016/j.molcel.2019.05.032 31226276

[65] Hsieh Y-YP, Makrantoni V, Robertson D, Marston AL, Murray AW. Evolutionary repair: changes in multiple functional modules allow meiotic cohesin to support mitosis. PLoS Biol. 2020;18:e3000635. doi: 10.1371/journal.pbio.3000635 32155147 PMC7138332

[66] Akematsu T, Fukuda Y, Garg J, Fillingham JS, Pearlman RE, Loidl J. Post-meiotic DNA double-strand breaks occur in *Tetrahymena*, and require Topoisomerase II and Spo11. Elife. 2017;6:e26176. doi: 10.7554/eLife.26176 28621664 PMC5482572

[67] Marcon L, Boissonneault G. Transient DNA strand breaks during mouse and human spermiogenesis: new insights in stage specificity and link to chromatin remodeling. Biol Reprod. 2004;70:910–8. doi: 10.1095/biolreprod.103.022541 14645105

[68] Calderwood SK. A critical role for topoisomerase IIb and DNA double strand breaks in transcription. Transcription. 2016;7:75–83. doi: 10.1080/21541264.2016.1181142 27100743 PMC4984685

[69] Wong MM, Belew MD, Kwieraga A, Nhan JD, Michael WM. Programmed DNA breaks activate the germline genome in *Caenorhabditis elegans*. Dev Cell. 2018;46:302–15. doi: 10.1016/j.devcel.2018.07.002 30086301

[70] Ju BG, Lunyak VV, Perissi V, Garcia-Bassets I, Rose DW, Glass CK, et al. A topoisomerase IIβ-mediated dsDNA break required for regulated transcription. Science. 2006;312:1798–902. doi: 10.1126/science.1127196 16794079

[71] Maynard Smith J. A short-term advantage for sex and recombination through sib-competition. J Theor Biol. 1976;63:245–58. doi: 10.1016/0022-5193(76)90033-3 1011844

[72] Douge M, Iwasa Y. Sibling diversity gives sexual reproduction the advantage in a changing environment. Evol Ecol Res. 2017;18:459–75.

[73] Karrer KM. *Tetrahymena* genetics: two nuclei are better than one. In: Asai DJ, Forney JD, editors. Tetrahymena thermophila. San Diego: Academic Press; 2000. p. 127–186. 10.1016/s0091-679x(08)61529-0 10503190

[74] Orias E, Singh DP, Meyer E. Genetics and epigenetics of mating type determination in *Paramecium* and *Tetrahymena*. Annu Rev Microbiol. 2017:133–56. doi: 10.1146/annurev-micro-090816-093342 28715961

[75] Chalker DL, Meyer E, Mochizuki K. Epigenetics of ciliates. Cold Spring Harb Perspect Biol. 2013;5:a017764. doi: 10.1101/cshperspect.a017764 24296171 PMC3839606

[76] Hamilton EP, Kapusta A, Huvos PE, Bidwell SL, Zafar N, Tang H, et al. Structure of the germline genome of *Tetrahymena thermophila* and relationship to the massively rearranged somatic genome. Elife. 2016;5:e19090. doi: 10.7554/eLife.19090 27892853 PMC5182062

[77] Eisen JA, Coyne RS, Wu M, Wu D, Thiagarajan M, Wortman JR, et al. Macronuclear genome sequence of the ciliate *Tetrahymena thermophila*, a model eukaryote. PLoS Biol. 2006;4:1621–42. doi: 10.1371/journal.pbio.0040286 16933976 PMC1557398

[78] Mochizuki K, Fine NA, Fujisawa T, Gorovsky MA. Analysis of a *piwi*-related gene implicates small RNAs in genome rearrangement in *Tetrahymena*. Cell. 2002;110:689–99. doi: 10.1016/s0092-8674(02)00909-1 12297043

[79] Sugai T, Hiwatashi K. Cytologic and autoradiographic studies of the micronucleus at meiotic prophase in *Tetrahymena pyriformis*. J Protozool. 1974;21:542–8. doi: 10.1111/j.1550-7408.1974.tb03695.x 4214068

[80] Mochizuki K, Gorovsky MAA. Dicer-like protein in *Tetrahymena* has distinct functions in genome rearrangement, chromosome segregation, and meiotic prophase. Genes Dev. 2005;19:77–89. doi: 10.1101/gad.1265105 15598983 PMC540227

[81] Bhalla N. Meiosis: is spermatogenesis stress an opportunity for evolutionary innovation? Curr Biol. 2020;30:R1471–3. doi: 10.1016/j.cub.2020.10.042 33352126

